# Association of ADHD With Congenital Conditions – Case Reviews in General Adult Clinic

**DOI:** 10.1192/bjo.2022.365

**Published:** 2022-06-20

**Authors:** Dipayan Roy

**Affiliations:** NHS Lanarkshire, Glasgow, United Kingdom

## Abstract

**Aims:**

The association of ADHD with mental health and medical conditions is commonly encountered in clinical practice. Interestingly there are patients with congenital conditions who present with features of ADHD and little is known about their association and neurological basis. There is no strong literature but anecdotal reports that indicate children with congenital heart disease are more likely to suffer from mental health conditions including ADHD. The clinic however is unable to analyze such hypothesis and instead decided to evaluate cases related to Neurofibromatosis (NF1), Arnold Chiari Malformation, Transposition of great arteries, Di George syndrome to understand the longitudinal history, symptom persistence and functional impact of ADHD.

**Methods:**

- Index patient aged 45 years referred for possible association of ADHD and Neurofibromatosis with issues related to long-standing trouble with sleep and movement disorder.
- Index patient aged 41 years received a surgical repair for a Chiari malformation hoping it would improve the cognitive functioning but still suffers lot of symptoms that are consistent with clinical picture of ADHD.
- Index patient aged 19 years referred for ADHD assessment reported history of transposition of great arteries and VSD that warranted emergency operative procedure before age 3. The behavioural symptoms that were suspected as related to physical illness and frequent attendance to hospital however did not resolve and were noted to be in line with possible ADHD.
- Index patient aged 40 years admitted to general psychiatry following episode of psychosis and during examination presented history of Di George syndrome with brief input from Cardiology. It was apparent that patient struggled with poor understanding, lack of consistency, disorganization, distractibility, learning difficulties and the features suggested a pattern of Attention deficit disorder.

**Results:**

Focused on

1. The qualitative analysis of developmental history, childhood rating scale, symptom comorbidity and functional impairment of such cases.
2. It studied the family history of physical and mental illness including predisposition to ADHD or neurodevelopmental conditions.
3. It also evaluated the treatment response to stimulant/non-stimulant therapy.

**Conclusion:**

Clinically there was no typical co-relation of increased mental illness or genetic predisposition for ADHD in the family history and qualitatively the presentation did not differ from other ADHD patients and the treatment response was not variable, however it still draws attention towards the need for regular screening of all nervous and cardiac origin congenital conditions for an early intervention.

